# 

**Published:** 1820-03

**Authors:** 


### TO SUBSCRIBERS.

*We have been obliged to omit the subjects ordinarily compressed in the department for* Medical Intelligence *in the present Number, from the necessity for the insertion of the last series of Papers; but we shall make amends for the deficiency here indicated in the next Number.*

